# The contraceptive potential of *Carica papaya* seed on oestrus cycle, progesterone, and histomorphology of the Utero-ovarian tissue of adult wistar rats

**DOI:** 10.5935/1518-0557.20200023

**Published:** 2021

**Authors:** Adejoke Elizabeth Memudu, Tayo Jane Oluwole

**Affiliations:** 1 Department of Anatomy, Faculty of Basic Medical Sciences, College of Medical Sciences, Edo University Iyamho, Edo State, Nigeria; 2 Department of Anatomy, College of Medicine, Bingham University; P.M.B 005, Karu, Nasarawa State, Nigeria

**Keywords:** *Carica papaya*, progesterone, utero-ovarian tissue, oestrus cycle

## Abstract

**Objective:**

The study’s goal was to ascertain the contraceptive effects of Aqueous extract of *Carica papaya* on female rats by assessing changes in the body weight, estrous cycle, serum progesterone level and the cyto-architecture of the Utero-ovarian tissue.

**Methods:**

We used twenty (20) healthy young Adult Female Albino rats. The study ran for 7 and 21 days, respectively. Each study group has their Experimental (treated 200mg/kg aqueous extract of *Carica papaya* seed extract) and Control group (n=5). We determined daily the phases and frequencies of the estrous cycles of the rats during the administration of the extract. We processed the utero-ovarian tissue for histological analysis, and we assessed serum progesterone level and the oestrus cycle pattern.

**Results:**

There was a significant increase in body and Ovarian weights after 21 days of treatment when compared to controls and those treated for 7 days. However, uterine weight reduced significantly (*p*<0.05), serum progesterone level decreased (*p*<0.05) in the treated rats, mostly in those submitted to 21 day-treatments; the ovary showed marked degeneration of the theca cells, granulosa and corpus luteum, and loss of mucin granules in the uterine tissues. Carica *papaya* administered for 7 and 21 days caused the animals to have more proestrus and diestrus phases as compared to the control animals. The estrous cycle became irregular, with prolonged diestrous and proestrus phase.

**Conclusion:**

The aqueous extract of Carica papaya seeds caused antifertility, anti-implantation, by a reduction in progesterone level, disruption of oestrus pattern and histological alteration of utero-ovarian tissue.

## INTRODUCTION

Over the years, the whole *Carica papaya* plant parts, leaves, seeds, ripe and unripe fruits, and their juice have been used as traditional medicine due to their high antioxidant activities ^(Arvind et al., 2013; Sindhu et al., 2019)^. Papaya juice or the fruit is generally served either green or ripe; while its leaves and young stems are sometimes steamed and served as a vegetable ^(Arvind *et al*., 2013)^. Nowadays, papaya (*Carica papaya*, Linn) is considered as a nutraceutical fruit, because of its exceptional nutritional and multifaceted medicinal properties ^(Parle & Guditta, 2011; Sindhu *et al*., 2019)^ which include antibacterial, anti-inflammatory, anti-aging, anti-proliferative, diuretic, anti-hypertensive, hypolipidemic, anti-helminthic, wound healing, anti-fungal and anti-tumor ^(Sarker et al., 2010; Kaushal et al., 2015; Gunde & Amnerkar, 2016; Sindhu *et al*., 2019)^ and free radical scavenging activities that helps to reduce atherosclerosis, strokes, diabetes, and heart attacks ^(Arvind *et al*., 2013)^. Phytochemically, the whole plant contains enzymes, lycopene, carotenoids, alkaloids, monoterpenoids, flavonoids, minerals and vitamins ^(Sindhu *et al*., 2019)^. The seeds have fatty acids, protein, fiber, papaya oil, sinigrin, Carpaine, benzyl isothiocyanate, benzyl glucosinolate, glucotropacolin, benzylthiourea, hentriacontane, β-sitosterol, caricin and an enzyme called myrosin ^(Azarkan et al., 2003; Adebiyi et al., 2002)^, while the *Carica papaya* leaves has alkaloids, flavonoids, saponins, tannins, cardiac glycosides, anthraquinones and cardenolides ^(Arvind *et al*., 2013; Sindhu *et al*., 2019)^. *Carica papaya* black seeds are edible, having sharp and spicy taste; hence, its ground form is used as an alternative for black pepper ^(Sindhu *et al*., 2019)^. Studies have reported that these seeds have more potent medicinal properties as compared to other parts of the papaya plant due to their strong antioxidant properties ^(Zhou et al., 2011; Fatima & Shahid, 2018; Otsuki et al., 2010; Sindhu *et al*., 2019)^; for example we have antibacterial (*E.coli*, Salmonella and Staphylococcus infections), liver protection, anthelminthic, typhoid treatments (Sindhu *et al*., 2019), nephroprotective effects ^(Otsuki *et al*., 2010)^ and in folk medicine it is an intestinal worm expellant in humans and ruminants.

Medicinal plants have played an important role in new drug developments. Birth control, also known as contraception and fertility control, is a method or device used to prevent pregnancy ^(Taliaferro et al., 2011)^. According to ^Westwood (2008)^ and ^Yuan & Foley (2002)^ drugs and chemical agents can interfere with the normal functions of the female reproductive cycle as well as interfere in the normal morphology of the reproductive organs. Studies have documented the role of medicinal plants in inducing infertility in experimental animals, and their possible development into contraceptive agents in males and females has gained continuous global attention by the pharmaceutical industry. Extracts of *Quassia amara*, *Azadirachta indica*, gossypol, a phenolic compound isolated from cottonseeds and glycosides extracted from xylem of *Tripterygium wilfordi*, appears to have been well-studied plant extracts for the induction of reversible infertility in male and female animals ^(Cherian, 2000)^.

*Carica papaya* seed extract has been associated with antifertility, documented by various studies in a quest to discover male contraceptive agents ^(Lohiya & Goyal, 1992; Lohiya *et al*., 1994; 1999; 2002; 2005; Chinoy et al., 1995; Pathak et al., 2000; Kusemiju et al., 2002; Udoh et al., 2005)^. Studies have reported antifertility potentials of plant parts of *Carica papaya* fruits and leaves in isolates without actually explaining the key mechanistic processes of antifertility. Studies have documented the antifertility effects of pawpaw seeds on the male reproductive system ^(Udoh & Kehinde, 1999; Chinoy *et al*., 1995; Lohiya *et al*., 1999; Udoh *et al*., 2005)^. ^Adebiyi et al. (2002)^ reported the unsafe ingestion of these seeds during pregnancy. The action of *Carica papaya* seeds in female animals is largely a product of folk medicine and there are varying reports in the literature on its mechanism of action in causing infertility in females ^(Karunamoorthi et al., 2014)^.

Assessment of the reproductive (oestrus) cycle is a valid way to study animals exhibiting abnormal oestrus patterns due to exposure of any compound and that caused changes to normal patterns ^(Goldman et al., 2007; McLean et al., 2012; Cora et al., 2015)^. This study was undertaken to explore the impact and mechanism of action of aqueous extract of *Carica papaya* seeds on estrous cycle and fertility in female albino rats.

## MATERIALS AND METHODS

### Experimental Animals

There were twenty (20) healthy young Adult Female Albino rats, of average weight of 120g, used for this study. We procured them from the Ahmadu Bello University, Zaria, Kaduna state, Nigeria. They were kept in well-aerated cages in the Animal House of Anatomy Department, Bingham University, Karu Nigeria. They were cared for according to guidelines from the ^National Research Council (US) Institute for Laboratory Animal Research (2011)^ and the Canadian Council on Animal Care ^(CCAC, 1984)^. They were allowed two (2) weeks acclimatization in standard laboratory conditions [12 hrs dark: 12 hrs light cycle, room temperature and humidity 52%]. They had free access to water and pelleted rat feed (Ladokun Feeds, Ibadan). Approval from the departmental research review ethical committee was obtained before the experiments. Rats have a short oestrus cycle, of 4 to 5 days ^(Long & Evans, 1922; Freeman, 1988; Mandl, 1951)^ and this makes it a perfect animal to investigate the effects of *Carica papaya* on the female reproductive system ^(Marcondes et al., 2001; McLean et al., 2012)^.

### Oestrus Cycle study

The rats' oestrus cycles were studied daily according to ^Marcondes et al., (2001; 2002)^ and ^Hubscher et al. (2005)^'s method. We selected rats with consecutive similar oestrus cycles for the experiment. The estrous cycle in a female rat is characterized by four different phases, which are diestrus, proestrus, oestrus, and metestrus. This cyclical pattern is completed within 4 to 5 days and they are microscopically evaluated via vaginal smear ^(Nelson et al., 1982; Goldman et al., 2007; McLean et al., 2012; Gonzalez, 2016)^.

### Preparation of the Vaginal Smear

The vaginal smear was done between 8:00 - 9:00 a.m. and 4:00 - 6 p.m. We collected the vaginal fluid using a plastic pipette filled with 0.5 ml of normal saline (NaCl 0.9%). The tip of the pipette was gently inserted into the rat vaginal opening. We then placed the aspirated vaginal fluid on a microscopic glass slide. We used a different microscopic glass slide for each experimental animal examined. We deposited one drop of the vaginal fluid on a slide and examined the unstained vaginal smear histology using an Olympus Light Microscope at 10 and 40 x objective lenses ^(Naik et al., 2015)^. We examined the four phases, described by ^Goldman et al. (2007)^ and ^Byers et al. (2012)^ they were: diestrus (D), when the cells are predominantly leucocytes; proestrus (P), with numerous nucleated cells; the oestrus (E) phase is marked by the presence of large numbers of cornified epithelial cells and, the last phase, metestrus (M), characterized by scattered squamous epithelial cells and several neutrophils. We used this procedure daily to assess the effects of *Carica papaya* seed extract on the oestrus cycle, for 21 days.

### Plant Material and Aqueous extract preparation

**Collection of *Carica papaya* seed:** Twelve (12) mature ripe *Carica papaya* (Paw-Paw) fruits were bought at the Mararaba market, Karu, Nasarawa State, Nigeria. The collected samples were identified and authenticated by a botanist from the Forestry Research Institute of Nigeria, where the Voucher specimen were documented in the Herbarium (106879).

**Preparation of *Carica papaya* seed extract:** The pawpaw fruits were cut open and the wet seeds brought out. They were gently rinsed in running tap water to clean it, and then they were air-dried for two weeks at room temperature. The dried *Carica papaya* seeds were then pulverized into a fine powder using a blender. 240g of powdered *Carica papaya* seed were macerated in 1000 ml of hot water for thirty (30) minutes and stirred every 5 minutes to ensure it was well soaked. Thereafter, the *Carica papaya* seed solution was filtered using a clean filter paper (Whatman Filter Paper) and we collected the filtrate into a conical flask according to the method described by ^Adebiyi et al. (2002)^, ^Dosumu et al. (2008)^ and ^Naggayi et al. (2015)^. We refrigerated the aqueous solution in an airtight container. The final filtrate was completely oven-dried at a preset temperature of 60ºC for a week, producing a fine aromatic and chocolate color solid residue (the dry aqueous seed extract) according to ^Naggayi *et al*. (2015)^.

### Aqueous *Carica papaya* seed extract dosage and mode of administration

Each experimental animal was given 1ml of 200mg/kg aqueous extract of *Carica papaya* seed Extract daily for 7 and 21 days respectively, according to ^Ruiz-Luna et al. (2005)^ and ^Naik et al. (2015)^. ^Olagunju et al. (2009)^ reported that LD50 of Aqueous *Carica papaya* seed extract is greater than 200mg/kg when taken orally.

### Experimental Design

Twenty (20) proestrus rats were used for this study, according to recommendations from ^Raji *et al*. (2005)^. They were studied for 7 and 21 days, respectively; each with their control groups for the study period (n=5). The phases and frequencies of the estrous cycle of the rats were determined daily for 21 days during the extract administration.

### Experimental Animal Euthanasia

Twenty-four hours after the last drug administration, we measured their body weights and the Animals were euthanized through neck dislocation.

### Blood collection and Preservation for Serum Hormone Analysis

The blood samples were collected using a 5ml syringe via cardiac puncture into the left ventricle, before neck dislocation. The collected samples were transferred into blood specimen bottles, they were than centrifuge at 2,500 rpm for 5 minutes to obtain the sera, which were aliquoted into plain specimen cuvettes and kept in the freezer ready for Progesterone ELISA kit quantitative spectrophotometric analysis.

### Serum Progesterone Analysis

Their serum progesterone activity was quantified using the Progesterone Enzyme-Linked immunosorbent assay (ELISA) kit (BXE0661A), Fortress Diagnostic Ltd. UK). We performed the assay according to the instructional manual from the kit manufacturer.

### Ovary and Uterine Tissues Collection

We made an incision on the pelvic region using a scalpel and carefully traced and excised the uterus and ovaries using a fine forceps. We measured their wet weights and preserved the tissues in a specimen bottle containing 10% formaldehyde saline ready for histological processing, according to ^Bancroft & Gamble’s (2008)^ method.

### Histological Tissue Processing and Staining

We fixed the ovary and uterus tissues in 10% formol saline and processed it using a Leica automated tissue processor. The processed tissues were embedded using paraffin wax in a Leica Embedding Machine. The tissue blocks were then serially sectioned using Leica Rotatory Microtome set at 5µm. We then mounted the sectioned tissues using DPX on glass slides, ready for Hematoxylin and Eosin, and Periodic acid Schiff stain (PAS) for Mucin granules, according to ^Bancroft & Gamble’s (2008)^ method.

### Tissue Photomicrography and Analysis

We examined the tissue slides using an Olympus Light Microscopy, with an attached digital camera with x10 objectives. We viewed the tissues at x10, x40 but the digital picture was taken at x10.

### Statistical Analysis

We analyzed the data using the SPSS version 17.0, and expressed it as mean ± Standard error of the mean (SEM). We used the Student t-test to compare the data from both groups. The level of statistical significance (*) was set at *p*<0.05.

## RESULTS

### Effects of Aqueous *Carica papaya* seed extract on body and organ weights

**Figure 1:**
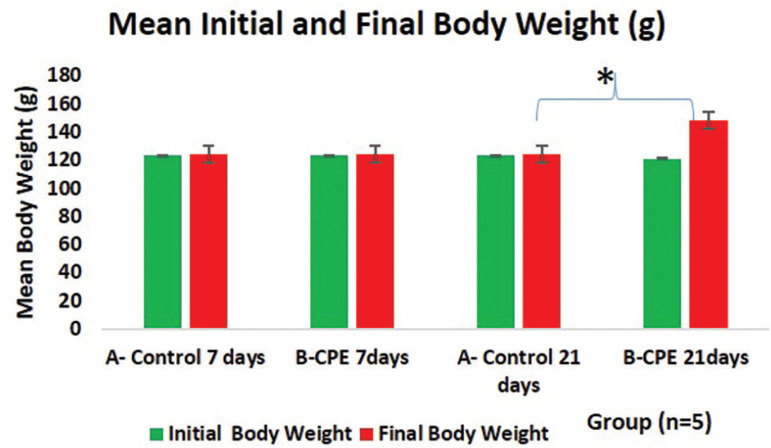
Graphical representation of the mean initial and final body weights of the experimental animals treated with 200mg/kg of aqueous *Carica papaya* seed extract. The asterisk (*) shows statistical significance (p<0.05). Legend: CPE = Carica papaya extract.

Oral administration of *Carica papaya* aqueous seed extract for 7 days had no significant effect on body weight gain when compared to the control animals (*p*<0.05) (Fig. 1). There was no significant difference between the initial and final weights of *Carica papaya* treated animals (*p*<0.05). However, we found that there was a significant increase in final body weight gain in those treated for 21 days when compared to their initial weights. There was a significant weight change between control and *Carica papaya*-treated groups during the 21 day-treatment study (Fig. 1).

### Aqueous *Carica papaya* seed extract reduced changes in ovary and uterus weights

**Figure 2:**
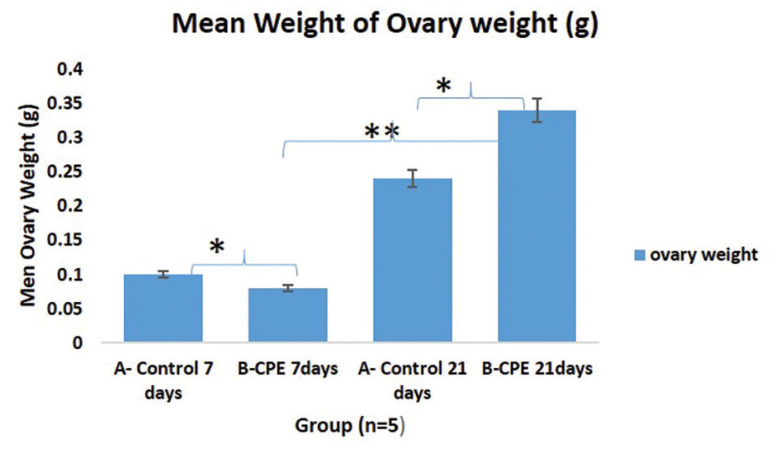
Graphical representation of the Mean Ovary Weight of the Experimental Animals treated with 200mg/ kg of aqueous *Carica papaya* seed extract. The asterisk (*) shows statistical significance (p<0.05). *: A vs. B; 7 days and A vs. B for 21 days; **: B 7 days treated vs. B 21-day treatment. Legend: CPE = *Carica papaya* extract

**Figure 3:**
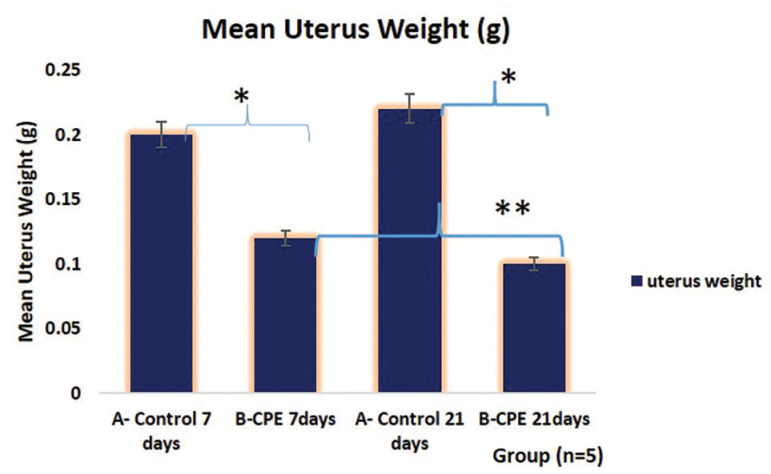
Graphical representation of the Mean Weight of the Uterus of Experimental Animals treated with 200mg/kg of aqueous *Carica papaya* seed extract. The asterisk (*) shows statistical significance (*p*<0.05). *: A vs. B; 7 days and A vs. B for 21 days; **: B 7 days treated vs. B 21 days treatment. Legend: CPE = *Carica papaya* extract

This study shows that aqueous extract of *Carica papaya* caused a statistically significant decrease in ovary weight following 7 days of treatment, as compared with its control group (Fig. 2). However, those treated for 21 days had a statistically significant increase in ovarian weight when compared with the Control animals for 21 days, and ACPSE treated for 7 days. As shown in Fig. 3, there was a significant reduction in the uterus’s wet weight in 7 and 21 day-treatments as compared with the control group (*p*<0.05).

### Aqueous *Carica papaya* seed extract decreases serum progesterone level

**Figure 4:**
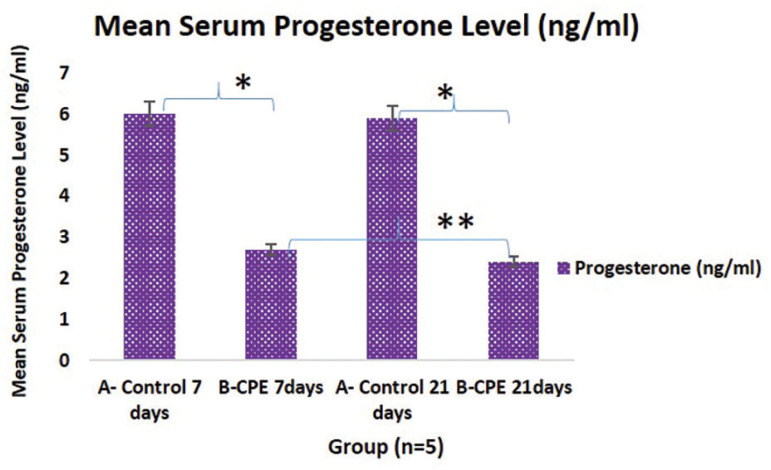
Graphical representation of the Mean Serum Progesterone level of Experimental Animals treated with 200mg/kg of aqueous *Carica papaya* seed extract. The asterisk (*) shows statistical significance (*p*<0.05). *: A vs. B; 7 days and A vs. B for 21 days; **: B 7 days treated vs. B 21 days treatment. Legend: CPE = *Carica papaya* extract

This study shows that the *Carica papaya* aqueous extract caused a statistically significant decrease in the serum progesterone level (*p*<0.05) in treated rats as compared with their control rats in their proestrus phase of the cycle (Fig. 4). Those treated for 21 days had a significant (**) reduction in progesterone levels when compared to the 7-day treatment group.

### Effects of aqueous *Carica papaya* seed extract on of oestrus cycle’s phases:

The control rats showed a regular and normal alternating cycle of diestrous, proestrus, estrus and metestrus phases. However, the *Carica papaya* seed extract-treated animals showed an extended pattern in the estrous cycle. The normal 4-5 day-cycles described by the study from ^Marcondes et al. (2002)^ was truncated in the *Carica papaya* study groups. *Carica papaya*-treated animals for 7 and 21 days had more Proestrus and diestrus phases as compared to their control counterparts. In this study, we found that the diestrus phase was dominant in the *Carica papaya* treatment as compared to the proestrus phase (Table 1).

**Table 1:** Changes in the Oestrus Cycles of the Experimental Animals treated with 200mg/kg of aqueous *Carica papaya* seed extract. Legend: CPE: Carica papaya extract; D: Diestrus phase; P: Proestrus phase; E: Estrus phase; M: Metestrus phase

A week's Oestrus Cycle during the administration of 200mg/kg of Carica papaya aqueous seed extract							
Groups (n=5)/days	Thursday	Friday	Saturday	Sunday	Monday	Tuesday	Wednesday
A-Control 7 days	D	P	E	M	D	D	P
B-CPE 7 days	P	P	P	D	D	D	P
							
Last week of administration - Oestrus Cycle - 200mg/kg of Carica papaya aqueous seed extract							
Groups (n=5)/days	Thursday	Friday	Saturday	Sunday	Monday	Tuesday	Wednesday
A-Control 21 days	M	E	D	D	P	E	P
B-CPE 21 days	P	P	D	D	D	D	D

### Effects of aqueous *Carica papaya* seed extract on ovarian tissue histology

**Figure 5:**
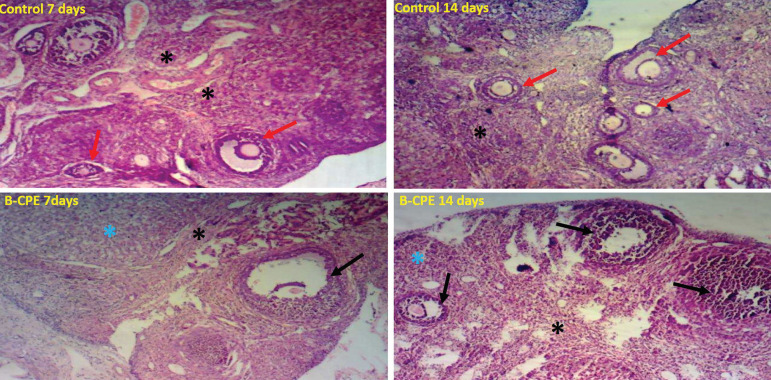
Micrograph of a cross-section of the Ovary of Experimental Animals treated with 200mg/kg of aqueous *Carica papaya* seed extract. Stained with Hematoxylin and Eosin (H and E stain). Mag x 100. The control group (A) at 7 and 14 days shows many healthy ovarian follicles in various stages of development. The theca and granulosa cells around the developing oocytes are well arranged, as compared to the *Carica papaya*-treated groups having few ovarian follicles, degeneration of the theca and granulosa cells of the ovarian follicles and corpus luteum (B- 7day-treatment) though mild as compared to 21-day treated *Carica papaya*. Legend: CPE = Carica papaya extract Ovarian stroma = black asterisk Corpus Luteum = blue asterisk Degenerated Follicles = black arrows Healthy follicle= red arrows

The *Carica papaya* study group had a distinctive lesion or degeneration of ovarian primordial and primary follicles as compared to the control group (Fig. 5). Follicular cells proliferation was grossly affected. Theca and granulosa cell arrangements were distorted as compared to the control group. The Corpus luteum appears degenerated and we noticed numerous striations/vacuolations in the ovarian stroma (Fig. 5).

### Effects of aqueous *Carica papaya* seed extract on uterine histoarchitecturee:

**Figure 6:**
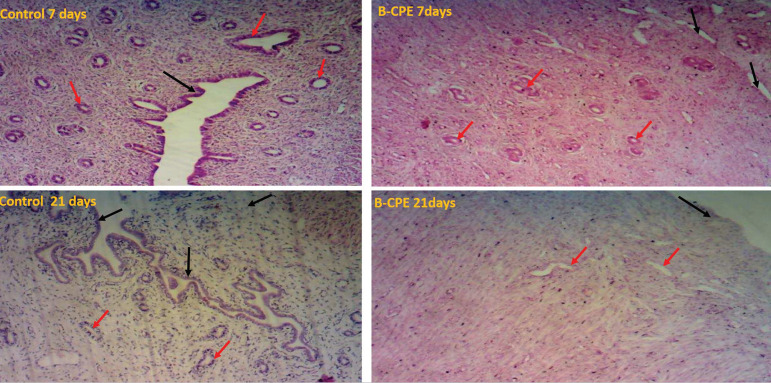
Micrograph of a cross-section of the Uterus of Experimental Animals treated with 200mg/kg of aqueous *Carica papaya* seed extract. Stained with Hematoxylin and Eosin (H and E stain). Mag x 100. The control group (A) at 7 and 21 days shows normal simple cuboidal epithelial cells lining the uterine gland, and well-pigmented endometrial cells as compared to *Carica papaya*-treated groups. The severity of uterine cellular distortion has higher in those treated for 21 days. Legend: CPE = *Carica papaya* extract Uterine glands = red arrows Endometrial lining = black arrows

**Figure 7:**
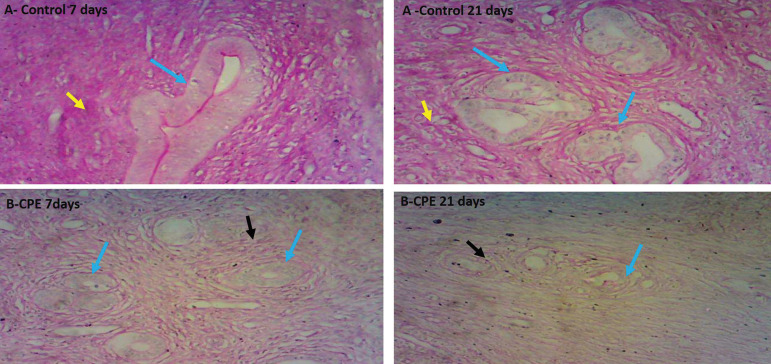
Micrograph of a cross-section of the Ovary of Experimental Animals treated with 200mg/kg of aqueous *Carica papaya* seed extract. Stained with Hematoxylin and Eosin (H and E stain). Mag x 100. The control group (A) at 7 and 14 days shows many healthy ovarian follicles at various stages of development. The theca and granulosa cells around the developing oocytes are well arranged, as compared to *Carica papaya*-treated groups, having few ovarian follicles, degeneration of the theca and granulosa cells of the ovarian follicles and corpus luteum (B- 7days treatment), though mild, as compared to 21 day-treated *Carica papaya*. Legend: CPE = *Carica papaya* extract Uterine glands = blue arrows Well stained mucin granules = yellow arrows Poorly stained mucin granules = black arrows

There was a gross loss of uterine tissue histological integrity in the *Carica papaya*-treated groups (Fig. 6). The uterine glands were poorly stained as compared to the control group with visible tubular uterine gland and well-pigmented endometrial lining. However, the severity of distortion or loss of cellular integrity was more often seen in the 21-day treated groups displaying severe loss of cellular pigmentation. Periodic Acid Stained Uterus demonstrated mucin granules, showing that the control groups were positive for PAS-stained structures, being positive for the presence of Mucin granules while the *Carica papaya*-treated groups (Fig. 7) were not positive for the mucin granules, indicating that the *Carica papaya* extract caused severe loss of mucin granules in the epithelial cells cytoplasm.

## DISCUSSION

The results obtained from this study showed that the aqueous extract of *Carica papaya* seed has a severe adverse effect on the oestrus cycles, progesterone level and the uterus and ovary histology. These aforementioned results justify its reported use as an abortifacient agent ^(Dosumu et al., 2008; Raji *et al*., 2005; Ekhator & Shelu, 2015)^.

*Carica papaya* prolonged intake caused an increase in body weight gain. However, the organ weight had varying effects; the ovary recorded an increase in weight while the uterus had a decrease in weight gain. *Carica papaya* caused reduction in testicular weight with no significant change in body weight ^(Lohiya et al., 1994)^. The aqueous extract of *Carica papaya* had a significant effect on body weight in this study. Body weight plays an important role in the regulation of gonadotropin secretion, and its crucial role in menstrual cycle regulation is well established. Microscopic evaluation of the estrous cycle in laboratory rats and mice is an index to estimate the functional status of the hypothalamic-pituitary-ovarian axis; it serves as a medium to evaluate changes in the estrous pattern when studying a compound to evaluate the effects on female reproductive function ^(Goldman et al., 2007; Cora et al., 2015)^.

*Carica papaya* increased the length of oestrus cycles and the frequency of diestrus. The diestrus phase persistence indicates that the extract interfered with the hypothalamic-pituitary-ovarian axis function, as seen in our study ^(Goldman et al., 2007)^. Through the gonadotropin hormones (FSH and LH), the pituitary-gonadal axis controls the pattern and frequency of the oestrus cycling ^(Cora et al., 2015)^. This abnormal cycling caused by *Carica papaya* seed was also reported by ^Raji *et al*. (2005)^, ^Dosumu et al. (2008)^, ^Odirichukwu et al. (2016)^ and ^Ganguly et al. (2007)^ studies on the *Mimosa pudica* root extract, and the ^Taid et al. (2016)^ study on *Plumeria acuminate*; thereby herbal plants can change oestrus cycles, indicating a potent anti-fertility effect. The root cause is linked to phytochemicals such as alkaloids, flavonoids, saponins, glycosides and phenolic compounds, having a chemical activity similar to reproductive hormones; hence mimicking hormone-like functions ^(Verma et al., 2016; Soni et al., 2016)^. Studies on alkaloids, flavonoids, saponins, and phenolic isolates reported they disrupt the oestrus patterns in animals ^(Ifeanyi et al., 2011; Verma *et al*., 2016; Soni *et al*., 2016; Fatima & Shahid, 2018)^.

The female estrous cycle is the time between periods of sexual receptivity. In mice and rats, this cycle is completed in 4/5-day intervals and reoccurs after that. The results obtained from this study on the effect of *Carica papaya* on the estrous cycle of female albino rats showed that the extract caused a significant alteration in the duration of the different phases of the estrous cycle ^(Taid et al., 2016)^. The normal process of the cycle as displayed by the control group was replaced by more presentation of the diestrus phase, which was prolonged. In addition, the numbers of cycles was reduced. Estrous is the heat period or mating phase, its duration was altered, as shown in our study, which suggests some antifertility effects of *Carica papaya*.

Hence, a reduction in the frequency of oestrous cycles from this study corresponds to a decrease in the rate of ovulation, which occurs from the beginning of pro-estrous to the end of oestrus ^(Young et al., 1941; Schwartz, 1964)^, resulting in decreased fertility ^(Nayaka et al., 2014)^. Progesterone plays an important role in influencing cycle length in rodents. It works in synergy with estrogen ^(Morali & Beyer, 1979)^. ^vom Saal et al. (1994)^ mentioned that the elevation of progesterone occurs before ovulation in young rats, but its decrease is linked to prolonging oestrus cycle length. Fluctuations in circulating levels of the ovarian steroids estradiol and progesterone, follicle-stimulating hormone and luteinizing hormone affects the estrous cycle and histological appearance of female reproductive organs ^(Lerner, 1969; McLean et al., 2012; Aritonang et al., 2017)^.

This present study shows that *Carica papaya* caused a significant decrease (*p*<0.05) in the serum level of progesterone. This indicates a normal function of progesterone which is the endometrial stromal fibroblast differentiation, uterine gland secretion and proliferation of uterine myometrium ^(Aritonang et al., 2017)^ is disrupted. A similar report was documented in male rats, where *Carica papaya* reduced in serum FSH and testosterone ^(Ansah et al., 2016)^. According to ^Biswal (2014)^, the level of estrogen and progesterone reaches its peak during proestrus, and a decline results in unbalanced ovarian functions, which invariably affect the estrus cycle and fertility index. The lowered level of progesterone seen is possibly linked to disruption in follicular development and absence of corpus luteum (a postovulatory follicle), that secretes progesterone, responsible for preparing the endometrium for the implantation of the fertilized ovum ^(Hadley, 1975; Pessina et al., 2006; Gonzalez, 2016)^. The loss of mucin granules and uterine gland proliferation seen in PAS stained uterus (Fig. 7) correlates with reports from ^Shapiro et al. (1980)^, a study in endometrium culture reporting that progesterone is linked with glycogen buildup in endometrial tissue, hence its reduction will reduced glycogen. Our study shows that *Carica papaya* induced a decrease in progesterone, resulting in the loss of glycogen/mucin granules in the endometrium. There were mild distortions, degeneration of follicular cells in the ovary, and the uterine cells were lightly stained, correlating with reports from ^Raji *et al*. (2005)^.

The significant structural morphological alterations seen in the histological sections of the *Carica papaya* seed extract-treated rats showed that the histological variation seen is associated with hormonal changes that also effect the oestrus cycle, explaining its antifertility effects. This extract induced severe degeneration of the follicular wall of the ovarian follicles, which explains the possible decrease in progesterone secretion (Fig. 4), and this resulted in anovulatory cycles. The hormonal and histological changes could lead to a significant reduction in ovarian and uterine weight (Figs. 2 and 3). ^Chinoy et al. (1994)^ showed that *Carica papaya* seed aqueous extract from 7 and 15 days caused more effects on the reproductive physiology in the ovary, as compared to the uterus, in addition to irregular oestrus cycle, characterized by a predominance of dioestrus. In this present study, we had severity in the uterus, contrary to the report from ^Chinoy *et al*. (1994)^.

The present study reports on the effect of Aqueous extract of *Carica papaya* on progesterone mechanistic secretion and influence on oestrus cycle and utero-ovarian histological appearance. The exact principles that caused the irregularity in the estrous cycle and infertility in *Carica papaya*-treated rats has not been fully documented. The fertility index is linked to the rate of ovulation ^(Young et al., 1941; Schwartz, 1964)^. *Carica papaya* decreases the rate of ovulation due to a prolonged diestrus phase; thereby, resulting in decreased fertility ^(Poharkar et al., 2010; Nayaka et al., 2014; Naik et al., 2015)^. It has been scientifically demonstrated that *C. papaya* seeds possess contraceptive and abortifacient activities. Our studies on the aqueous seed extract also showed reduced fertility, evidenced by irregular estrous cycle, reduction of progesterone activity, loss of mucin granules and an indication of uterine tubular glands secretory activity. The proestrus phase of the estrous cycle corresponds to the human follicular phase of the menstrual cycle with elevated FSH concentrations. This mechanism of action, illustrated from results obtained from this present study, supports abortifacient, anti-implantation linked to female infertility ^(Poharkar *et al*., 2010; Ansah et al., 2016)^. This gives an indication that the *Carica papaya* seed extract may act on the ovary through altered endocrine pathways, associated with decreased progesterone levels. In addition to decreasing infertility, papain might cause abortions shortly after conception. Papain dissolves proteins responsible for the newly fertilized egg adhering to the uterine wall. ^Goyal et al. (2010)^ carried out a study to authenticate scientific documentation of the papaya seeds being traditionally used for contraception, and establishing the safety of the papaya seeds methanol sub-fraction (MSF) as a male contraceptive following long term oral intake.

## CONCLUSION

The *Carica papaya* seed extract shows antifertility, anti-implantation and abortifacient effects in the treated animals associated with its ability to alternate hormonal activity that regulates the estrous cycle. Hence, it can be use as a contraceptive agent.

## References

[r1] Adebiyi A, Adaikan PG, Prasad RN (2002). Papaya (Carica papaya) consumption is unsafe in pregnancy: fact or fable? Scientific evaluation of a common belief in some parts of Asia using a rat model. Br J Nutr..

[r2] Ansah C, Appaih JA, Mensah KB, Mante PH (2016). Aqueous leaf extract of Carica papaya (Caricaceae) Linn. causes liver injury and reduces fertility in rats. Int J Pharm Pharm Sci.

[r3] Aritonang TR, Rahayu S, Sirait LI, Karo BR, Simanjuntak TP, Natzir R, Sinrang AW, Massi NM, Hatta M, Kamelia E (2017). The role of FSH, LH, estradiol and progesterone hormone on estrous cycle of female rats. Int J Sci Basic Appl Res.

[r4] Arvind G, Bhowmik D, Duraivel S, Harish G (2013). Traditional and medicinal uses of Carica papaya. J Med Plants Stud.

[r5] Azarkan M, El Moussaoui A, van Wuytswinkel D, Dehon G, Looze Y (2003). Fractionation and purification of enzymes stored in the latex of Carica papaya. J Chromatogr B Analyt Technol Biomed Life Sci.

[r6] Bancroft DD, Gamble M (2008). Theory and Practice of Histologic Techniques.

[r7] Biswal S (2014). Phytochemical analysis and a study on the antiestrogenic antifertility effect of leaves of Piper betel in female albino rat. Anc Sci Life..

[r8] Byers SL, Michael MV, Dunn SL, Taft RA (2012). Mouse estrous cycle identification tool and images. PLoS One.

[r9] CCAC - Canadian Council on Animal Care (1984). Guide to Care and Use of Experimental Animals.

[r10] Cherian T (2000). Effect of papaya latex extract on gravid and non- gravid rat uterine preparation in vitro. J Ethnopharmacol.

[r11] Chinoy NJ, D'Souza JM, Padman P (1994). Effects of crude aqueous extract of Carica papaya seeds in male albino mice. Reprod Toxicol.

[r12] Chinoy NJ, Dilip T, Harsha J (1995). Effect of Carica papaya seed extract on female rat ovaries and uteri. Phytother Res..

[r13] Cora MC, Kooistra L, Travlos G (2015). Vaginal Cytology of the Laboratory Rat and Mouse: Review and Criteria for the Staging of the Estrous Cycle Using Stained Vaginal Smears. Toxicol Pathol.

[r14] Dosumu OO, Akinola OB, Oremosu AA, Noronha CC, Okanlawon AO (2008). Antifertility Effects of the Aqueous Extract of Carica Papaya (Linn.) Seeds on Estrous Cycle and Ovulation of Adult Cyclic Sprague-Dawley Rats. Niger J Health Biomed Sci.

[r15] Ekhator CN, Shelu JO (2015). An Experimental Study on the Abortificient Potentials of Unripe Seed Extract of Carica papaya in adult Female Wistar Rats. Open Sci J Pharm Pharmacol.

[r16] Fatima U, Shahid S (2018). Pharmacological Activities of Carica papaya Linn. J Basic Appl Sci.

[r17] Freeman ME, Knobil E, Neil J (1988). The neuroendocrine control of the ovarian cycle of the rat. Physiology of Reproduction.

[r18] Ganguly M, Devi N, Mahanta R, Borthakur MK (2007). Effect of Mimosa pudica root extract on vaginal estrous and serum hormones for screening of antifertility activity in albino mice. Contraception.

[r19] Goldman JM, Murr AS, Cooper RL (2007). The rodent estrous cycle: characterization of vaginal cytology and its utility in toxicological studies. Birth Defects Res B Dev Reprod Toxicol.

[r20] Gonzalez G (2016). Determining the Stage of the Estrous Cycle in Female Mice by Vaginal Smear. Cold Spring Harb Protoc.

[r21] Goyal S, Manivannan B, Ansari AS, Jain SC, Lohiya NK (2010). Safety evaluation of long term oral treatment of methanol sub-fraction of the seeds of Carica papaya as a male contraceptive in albino rats. J Ethnopharmacol.

[r22] Gunde MC, Amnerkar ND (2016). Nutritional, medicinal and pharmacological properties of papaya (Carica papaya linn.): A review. J Innov Pharm Biol Sci.

[r23] Hadley JC (1975). Total unconjugated oestrogen and progesterone concentrations in peripheral blood during the oestrous cycle of the dog. J Reprod Fertil.

[r24] Hubscher CH, Brooks DL, Johnson JR (2005). A quantitative method for assessing stages of the rat estrous cycle. Biotech Histochem.

[r25] Ifeanyi AC, Yama OE, Ikechukwu DF, Adewale OA, Noronha CC, Olugbenga OA (2011). Effect of Momordica charantia on estrous cycle of Sprague-Dawley rats. Pac J Med Sci.

[r26] Karunamoorthi K, Kim HM, Jegajeevanram K, Xavier J, Vijayalakshmi J (2014). Papaya: A gifted nutraceutical plant - a critical review of recent human health research. TANG.

[r27] Kaushal VS, Verma KS, Ayachi A (2015). Capability of different plant parts of Carica papaya to scavenge peroxide free radical in vitro. World J Pharm Pharm Sci.

[r28] Kusemiju O, Noronha C, Okanlawon A (2002). The effect of crude extract of the bark of Carica papaya on the seminiferous tubules of male Sprague-Dawley rats. Niger Postgrad Med J.

[r29] Lerner LJ, Lednicer D (1969). The biology of non-steroidal antifertility agents. Contraception: The chemical control of fertility.

[r30] Lohiya NK, Goyal RB (1992). Antifertility investigations on the crude chloroform extract of Carica papaya Linn. seeds in male albino rats. Indian J Exp Biol.

[r31] Lohiya NK, Goyal RB, Jayaprakash D, Ansari AS, Sharma S (1994). Antifertility effects of aqueous extract of Carica papaya seeds in male rats. Planta Med.

[r32] Lohiya NK, Pathak N, Mishra PK, Manivannan B (1999). Reversible contraception with chloroform extract of the Carica papaya Linn. seeds in male rabbits. Reprod Toxicol.

[r33] Lohiya NK, Manivannan B, Mishra PK, Pathak N, Sriram S, Bhande SS, Panneerdoss S (2002). Chloroform extract of Carica papaya seeds induces long term reversible azoospermia in langur monkey. Asian J Androl.

[r34] Lohiya NK, Mishra PK, Pathak N, Manivannan B, Bhande SS, Panneerdoss S, Sriram S (2005). Efficacy trial on the purified compounds of the seeds of Carica papaya for male contraception in albino rat. Reprod Toxicol.

[r35] Long JA, Evans HM (1922). The oestrous cycle in the rat and its associated phenomena.

[r36] Mandl AM (1951). The phases of the oestrous cycle in the adult white rat. J Exp Biol.

[r37] Marcondes FK, Bianchi FJ, Tanno AP (2002). Determination of the estrous cycle phases of rats: some helpful considerations. Braz J Biol.

[r38] Marcondes FK, Miguel KJ, Melo LL, Spadari-Bratfisch RC (2001). Estrous cycle influences the response of female rats in the elevated plus-maze. Physiol Behav.

[r39] McLean AC, Valenzuela N, Fai S, Bennet SAL (2012). Performing vaginal lavage, crystal violet staining, and vaginal cytological evaluation for mouse estrous cycle staging identification. J Vis Exp.

[r40] Morali G, Beyer C, Beyer C (1979). Neuroendocrine control of mammalian estrous behavior. Endocrine control of sexual behavior.

[r41] Naggayi M, Mukiibi N, Iliya E (2015). The protective effects of aqueous extract of Carica papaya seeds in paracetamol induced nephrotoxicity in male wistar rats. Afr Health Sci.

[r42] Naik NR, Malleswari D, Indira P (2015). Effect of Mesua ferrea flower and Carica papaya seed extracts on estrous cycle of female albino rats for fertility and anti-fertility activity. Biolife.

[r43] National Research Council (US) Institute for Laboratory Animal Research (2011). Guidance for the Description of Animal Research in Scientific Publications.

[r44] Nayaka HB, Londonkar RL, Andumesh MK (2014). Evaluation of Portulaca oleracea for anti-fertility effect in female albino Rats. Int J Pharm Pharm Sci..

[r45] Nelson JF, Felicio LS, Randall PK, Sims C, Finch CE (1982). A longitudinal study of estrous cyclicity in aging C57BL/6J mice: I. Cycle frequency, length and vaginal cytology. Biol Reprod..

[r46] Odirichukwu EO, Uchechukwu NVS, Ogwu D (2016). The Aqueous Methanolic Extract of Unripe Carica papaya (Pawpaw) Fruit Distrupts Oestrous Cycle in Albino Rats. IOSR J Agric Vet Sci.

[r47] Olagunju JA, Adeneye AA, Fagbohunka BS, Bisuga NA, Ketiku AO, Benebo AS, Olufowobi OM, Adeoye AG, Alimi MA, Adeleke AG (2009). Nephroprotective activities of the aqueous seed extract of Carica papaya Linn. in carbon tetrachloride induced renal injured Wistar rats: a dose- and time-dependent study. Biol Med..

[r48] Otsuki N, Dang NH, Kumagai E, Kondo A, Iwata S, Morimoto C (2010). Aqueous extract of Carica papaya leaves exhibits anti-tumor and immunomodulatory effects. J Ethnopharmacol.

[r49] Parle M, Guditta A (2011). Basketful benefits of Papaya. Intern Res J Pharm.

[r50] Pathak N, Mishra PK, Manivannan B, Lohiya NK (2000). Sterility due to inhibition of sperm motility by oral administration of benzene chromatographic fraction of the chloroform extract of the seeds of Carica papaya in rats. Phytomedicine.

[r51] Pessina MA, Hoyt Jr RF, Goldstein I, Traish AM (2006). Differential effects of estradiol, progesterone, and testosterone on vaginal structural integrity. Endocrinology.

[r52] Poharkar RD, Saraswat RK, Kotkar S (2010). Survey of plants having anti-fertility activity from Western Ghat area of Maharashtra state. J Herb Med Toxicol.

[r53] Raji Y Morakinyo AO, Oloyo AK Akinsomisoye OS, Olufadekemi Kunle-Alabi T, Esegbue-Peters PRC Awobajo FO (2005). Impact of the Chloroform Extract of Carica papaya Seed on Oestrous Cycle and Fertility in Female Albino Rats. J Med Sci..

[r54] Ruiz-Luna AC, Salazar S, Aspajo NJ, Rubio J, Gasco M, Gonzales GF (2005). Lepidium meyenii (Maca) increases litter size in normal adult female mice. Reprod Biol Endocrinol.

[r55] Sarker SK, Begum N, Mondal D, Siddique A, Rashid MA (2010). In vitro study of antiamoebic effect of methanolic extract of mature seeds of Carica papaya on trophozoites of Entamoeba histolytica. Bangladesh J Pharmacol.

[r56] Schwartz NB (1964). Acute effects of ovariectomy on pituitary LH, uterine weight, and vaginal cornification. Am J Physiol.

[r57] Shapiro SS, Dyer SD, Colás AE (1980). Progesterone-induced glycogen accumulation in human endometrium during organ culture. Am J Obstet Gynecol.

[r58] Sindhu G, Ragini G, Sreehari L, Gayathri G, Thejaswee A, Inthiyaz S, Usha Kiran Reddy T, Thyagaraju K (2019). Pharmacognostical and pharmacological profile of carica papaya - A review. World J Pharm Pharm Sci.

[r59] Soni P, Siddiqui AA, Dwivedi J, Soni V (2016). Antiovulatory and estrogenic activity of leaves of Datura stramonium linn. in female albino rats. Asian J Pharm Res Health Care.

[r60] Taid TC, Rajkhowa RC, Kalita JC (2016). The effect of plumeria acuminata ait on oestrous cycle and acute oral toxicity study in c3h female albino mice. Int J Dev Res.

[r61] Taliaferro LA, Sieving R, Brady SS, Bearinger LH (2011). We have the evidence to enhance adolescent sexual and reproductive health--do we have the will?. Adolesc Med State Art Rev.

[r62] Udoh P, Essien I, Udoh F (2005). Effects of Carica papaya (paw paw) seed extract on the morphology of pituitary-gonadal axis in male Wister rats. Phytother Res..

[r63] Udoh P, Kehinde A (1999). Studies on the antifertility effect of pawpaw seeds (Carica papaya) on the gonads of male albino rats. Phytother Res..

[r64] Verma A, Pragya S, Singh VN (2016). Reversible Anti-Fertility Effects of Aqueous Leaf Extract of Ocimum Sanctum (Linn.) in Male Mice. Int J Life Sci Sci Res.

[r65] vom Saal FS, Finch CE, Nelson JF, Knobil E, Neil JD (1994). Natural history and mechanism of reproductive aging in humans, laboratory rodents, and other selected vertebrates. The Physiology of Reproduction.

[r66] Westwood FR (2008). The female rat reproductive cycle: a practical histological guide to staging. Toxicol Pathol.

[r67] Young WC, Boling JL, Blandau R (1941). The vaginal smear picture, sexual receptivity and time of ovulation in the albino rats. Anat Rec..

[r68] Yuan YD, Foley GL, Haschek WM, Rousseaux CG, Wallig MA (2002). Female reproductive system. Handbook of Toxicologic Pathology.

[r69] Zhou K, Wang H, Mei W, Li X, Luo Y, Dai H (2011). Antioxidant activity of papaya seed extracts. Molecules.

